# Analysis of Flow Cytometric Fluorescence Lifetime with Time-Delay Estimation of Pulse Signals

**DOI:** 10.3390/s18020442

**Published:** 2018-02-03

**Authors:** Lianqing Zhu, Wenchang Zhang, Mingli Dong, Xiaoping Lou

**Affiliations:** 1 Beijing Key Laboratory for Optoelectronics Measurement Technology, Beijing Information Science and Technology University, Beijing 100192, China; zhulianqing@sina.com (L.Z.); louxiaoping@bistu.edu.cn (X.L.); 2 Key Lab of Microelectronic Devices & Integrated Technology, Institute of Microelectronics, Chinese Academy of Sciences, Beijing 100029, China; zhangwenchang@ime.ac.cn

**Keywords:** flow cytometry, time-delay estimation, fluorescence lifetime, pulse width variation

## Abstract

The measurement of fluorescence lifetimes emerged in flow cytometry because it is not impacted by the non-linearity, which occurs in fluorescence intensity measurements. However, this significantly increases the cost and complexity of a traditional flow cytometer. This work reports a simple method of fluorescence lifetime measurement of a flow cytometer based on the cytometric fluorescence pulse time-delay estimation and hardware time-delay calibration. The modified chirp Z-transform (MCZT) algorithm, combined with the algorithm of fine interpolation of correlation peak (FICP), is applied to improve the temporal resolution of the cross-correlation function of the scattering and fluorescence signals, which in turn improves the time-delay estimation accuracy. The estimation accuracy is verified by Gauss fitting. Cells that were labeled simultaneously with three-color reagents are measured; the statistical results of 5000 cells are compared with reference values and are verified with the pulse width variation. The results show the potential of fluorescence lifetime measurements in the traditional flow cytometer.

## 1. Introduction

Flow cytometry (FCM) is an important clinical diagnostics technology used in immunology, hematology, oncology and basic biomedical research. Single cells or other microspheres in suspension move linearly through a flow chamber at a high speed and are illuminated by a shaped laser beam. By measuring the scatter light signal and the fluorescence signal, the sample microspheres can be quantitatively detected at a high speed, one by one. The measurement of fluorescence lifetimes is emerging in FCM, because it is not impacted by the non-linearity that occurs in intensity measurements. It adds in a variety of single-cell, multi-parametric measurements [1,2,3,4]. Moreover, fluorescence lifetime measurements by flow cytometer yield additional information about fluorophore–cell interactions at the molecular level. Fluorescence lifetime does not depend on initial perturbation conditions, such as light exposure duration, excitation wavelength, measurement method, single or multiphoton excitation and is not affected by photo bleaching. It could be considered as a state function. Moreover, fluorescence lifetime is independent of the fluorescence intensity and fluorophore concentration. It is rendered as a separate yet complementary method to traditional fluorescence intensity measurements [5,6,7,8].

The two main approaches of fluorescence lifetime detection that have been applied in flow cytometry are “frequency-domain” and “time-domain” methods. With frequency-domain method, the excitation laser is sinusoidally modulated at high frequencies, the fluorescence is emitted and oscillate at the same frequency as the excitation light. The modulated fluorescence light is amplitude-attenuated and phase-shifted with respect to the incident light [7,8]. In time-domain systems, the excitation source is typically pulsed at a femtosecond rate. The fluorescence lifetime is resolved by observing the fluorescence decay over time with a gated photo-detector and then fitted in an appropriate exponential-decay (single exponential or multi-exponential) model. The cost and complexity of a traditional flow cytometer increases significantly with both time- and frequency-domain approaches. Several high-frequency hardware components must be added to the electrical and signal processing systems to perform phase-sensitive flow cytometer. Some steps such as high-frequency mixing and filtering are introduced if the high-frequency signals are out of the range of data acquisition system employed [9,10,11,12,13,14,15,16,17,18]. 

This paper reports a simple fluorescence lifetime measurement method based on cytometric time-delay estimation of pulse signals and calibration of hardware time-delay. A high-resolution analysis method of the cross-correlation frequency spectrum based on MCZT algorithm is applied and then the FICP algorithm is integrated to improve the temporal resolution. Gauss fitting algorithm is introduced for the verification of time-delay estimation. The calculated lifetimes of three fluorescence pulse signals are compared with reference values provided by the vendors and with pulse width variations. This method avoids modulating the excitation source at high frequency and effectively presents a method for lifetime detection that could be implemented in most standard flow cytometers more practically.

## 2. Theory

### 2.1. Flow Cytometric Fluorescence Lifetime

**Figure 1:**
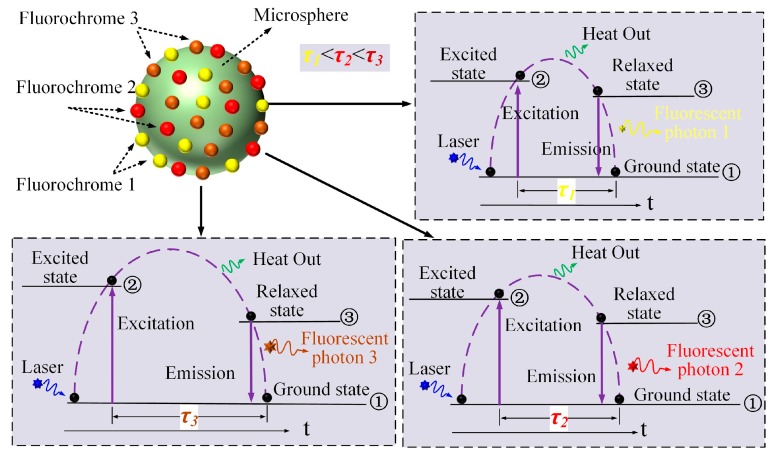
Schematic representation of fluorescence excitation process of the fluorescently labeled microsphere with three different fluorochromes.

Figure 1 shows a schematic representation of laser excited fluorescence of a fluorescently labeled microsphere (green sphere) with three types of fluorochrome (fluorochrome 1: yellow ball, fluorochrome 2: red ball, fluorochrome 3: brown ball). The microsphere is irradiated by the laser photon (blue star) and the outer electron (black ball) transited from the ground state ① to the excited-signal state ② and then relaxed to a single state ③ with some energy lost in the form of heat. Upon returning to ground state, a fluorescent photon (fluorescent photon 1: yellow star, fluorescent photon 2: red star, fluorescent photon 3: brown star) is emitted. Each of the processes occurs with a certain probability, characterized by decay rate constants (*k*). The fluorescence lifetime is defined as the average length of time for the set of molecules to decay from one ② to ①, which is inversely proportional to the rate of decay (*τ = 1/k*) [18,19,20,21]. The corresponding fluorescence lifetimes of the three types of fluorochrome in Figure 1 are marked as *τ*_1_, *τ*_2_ and *τ*_3_ (*τ*_1_ < *τ*_2_ < *τ*_3_).

As illustrated in Figure 1, fluorescence lifetime is determined by the electron energy level structure of the fluorochrome molecules in the specific surrounding microenvironment. However, since fluorescence lifetimes of different fluorochromes generally range from 0.1 ns to 20 ns, it is impossible to measure them directly with the standard flow cytometer. Considering that the scatter pulses are emitted as soon as the laser photons arrive at the microsphere, the time-delay between the scatter and fluorescence light pulse signals can be used to represent the lifetime.

### 2.2. Forward-Scattered and Fluorescent Light Pulses

**Figure 2:**
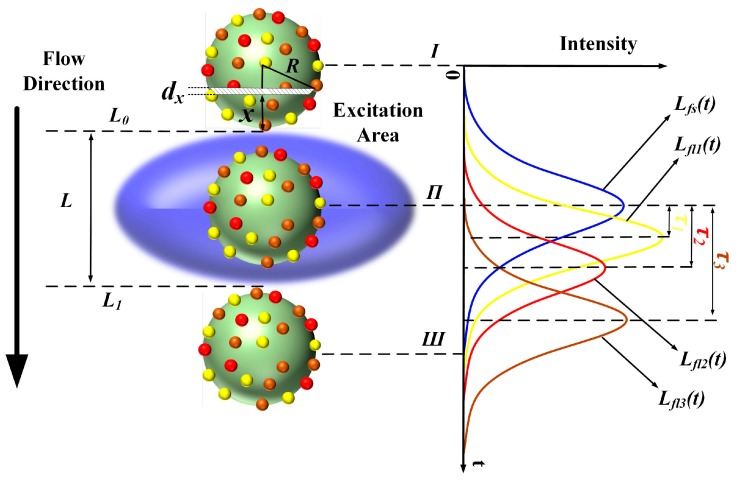
Generation of forward-scattered and fluorescent light pulses while microsphere flows through the excitation volume. (*L_fs_*(*t*): forward-scattered light pulse; *L_fl_*_1_(*t*), *L_fl_*_2_(*t*) and *L_fl_*_3_(*t*): light pulse of fluorochromes 1, 2 and 3, respectively; *L*_0_: and *L*_1_: the upper and lower limbs of excitation volume, respectively).

Qualitatively, the forward-scattered light pulse (*L_fs_*(*t*)) and fluorescent light pulses *L_fl_*(*t*) (*L_fl_*_1_(*t*), *L_fl_*_2_(*t*) and *L_fl_*_3_(*t*)) are generated when a microsphere flows through the laser excitation volume as shown in Figure 2. Conventionally, the laser beam is focused into elliptical excitation volume with the length of the minor axis slightly longer than the diameter of tested microspheres or cells. The microsphere moves along the flow direction through the excitation area (blue ellipsoid in Figure 2). A Gaussian-like trace reflects the resulting signal, where the forward-scattered emission begins to increase at time *Ι* (the microsphere flows across the upper limb *L*_0_ into the excitation volume), peaks at time *Π* and decreases as the microsphere moves from time *Π* to time *Ш* (the microsphere flows across the lower limb *L*_1_). 

Assume that the minor axis length of excitation volume is *L*, the radius of the microsphere is *R* and the microsphere flows at a constant speed *v*. Spatially, the laser intensity distribution along the flow direction could be assumed as $e^{-{\left(l-L/2\right)}^{2}/（2\sigma^{2}）}$, where σ is an invariant related to *L*. Therefore, the transit time is (*L* + *2R*)/*v*. The microsphere could be divided into *M* subintervals of equal width along the flow direction. Each subinterval could be considered as a cylinder, with the height defined as *dx* = *2R*/*M*. Therefore, at time *t* = 0 the distance between the *i*th subinterval and *L*_0_ is *x* = *i2R*/*M* and the lateral area of the *i*th subinterval is $2\pi \sqrt{R^{2}-{\left(R-x\right)}^{2}}dx$. At time *t* = *t*_0_, the *i*th subinterval arrives at the location where the laser intensity of the excitation volume is $e^{-{\left(vt_{0}-x-L/2\right)}^{2}/（2\sigma^{2}）}$. Finally, the time-dependent forward-scattered light intensity is given as:

(1) $$L_{fs}(t)=\int_{0}^{2R}2\pi \sqrt{R^{2}-{\left(R-x\right)}^{2}}e^{-\frac{{\left(vt-x-L/2\right)}^{2}}{2\sigma^{2}}}dx$$

where *t* ranges from 0 to (*L* + *2R*)/*v*.

The peak value and peak location of the cytometric pulses would be influenced by fluorescence lifetime *τ*. Some other influencing factors, such as the number of fluorochromes labelled on the cell and the quantum efficiency, are neglected here to analyze the individual influence of fluorescence lifetime. Assuming single exponential decay kinetics, the fluorescence light signal (*L_fl_*(*t*)) can be expressed as a convolution of the forward-scattered light and the single exponential decay, such that [22,23,24]:

(2) Lfl(t)=Lfs(t)*e−tτ

where symbol * means convolution operation.

Considering the frequency spectrum (*L_fl_*(*ω*)) of *L_fl_*(*t*) as follows:

(3) $$\begin{array}{c} L_{fl}(\omega )=L_{fs}(\omega )\cdot E(\omega )=A\cdot E\cdot e^{-j\varphi_{fs}}\cdot e^{-j\varphi_{E}}\\ =A\cdot E\cdot e^{-j\varphi_{fs}}\cdot e^{-j(\arctan\omega \tau )}\\ \approx A\cdot E\cdot e^{-j\varphi_{fs}}\cdot e^{-j\omega \tau } \end{array}$$

where *L_fs_*(*ω*), *E*(*ω*) is the frequency spectrum of *L_fs_*(*t*) and e−tτ, respectively; *A* and *φ_fs_* are the amplitude and phase of *L_fs_*(*ω*); *E* and *φ_E_* are the amplitude and phase of *E*(*ω*). The phase shift between *L_fs_*(*ω*) and *L_fl_*(*ω*) is $e^{-j\omega \tau }$, therefore, the time-delay between *L_fs_*(*t*) and *L_fl_*(*t*) is the fluorescence lifetime *τ*. Namely, the time-delay between peak locations of pulse signals is *τ*. As shown in Figure 2, the time-delays between the forward-scattered and the fluorescence pulse signals shown are *τ*_1_, *τ*_2_ and *τ*_3_. Due to the process of convolution, *τ* will cause the asymmetry of fluorescence pulse signals. However, compared with the pulse width, *τ* is small enough to neglect the asymmetry. The fluorescence pulses could be expressed as Gaussian shapes. Some results of Gauss fitting of fluorescence pulses could be found in Section 4.2.

**Figure 3:**
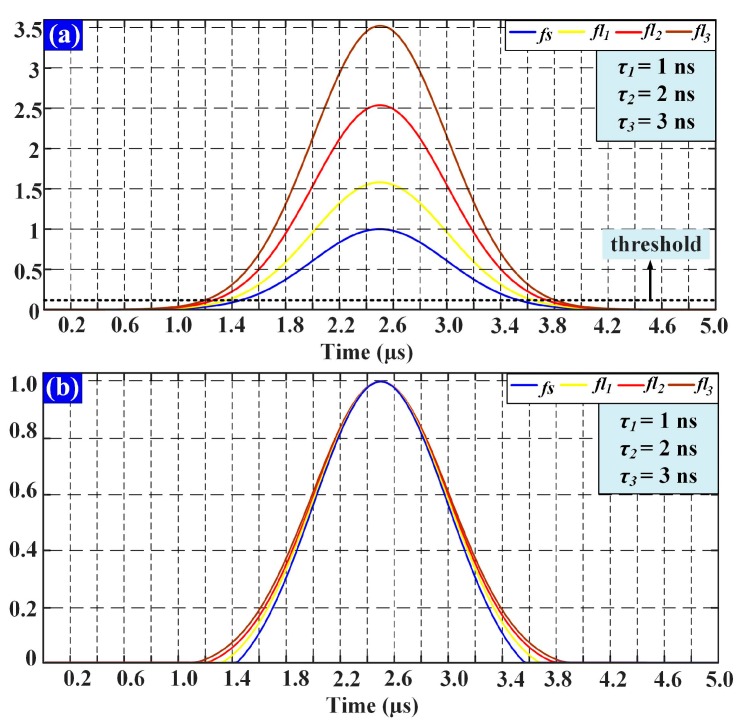
(**a**) The waveforms of fluorescent pulse signals with different lifetimes; (**b**) The change of pulse width of fluorescent signals with different lifetimes resulted from the threshold. (The intensity in this figure is used to represent relativeness with no units.)

In order to illustrate the influence of fluorescence lifetime, a Gaussian signal is taken as *fs*(*t*) for the simulation analysis. Considering that, the cytometric pulse width is about the microsecond level generally (depends on the sample flow rate, cell size and the minor axis length of excitation volume), the width of *fs*(*t*) is set at 5 μs. Three fluorescence pulse signals with different lifetimes (*τ*_1_ = 1 ns, *τ*_2_ = 2 ns, *τ*_3_ = 3 ns) calculated using Equation (2) are shown in Figure 3. 

As illustrated in Figure 3a, longer lifetimes will introduce higher peak values. Considering that, there is a sensitivity limit in the detectors (traditionally, PMT: photomultiplier tube), which are used to detect the fluorescence light. There will be a threshold for the detection. In other words, when the intensity is below the threshold, fluorescence light would not be detected. For this reason, the width of the fluorescence pulse signal is decreased, as shown in Figure 3b. The peak values of fluorescence pulse signals with different lifetimes are normalized to illustrate the change of pulse width. Taking a certain width of forward-scattered signal and a certain threshold value, the relationship between lifetime and width variation can be acquired. The width variation could be used for the verification of lifetime measurement accuracy. Moreover, as mentioned above, time-delays between the forward-scattered and the fluorescence pulse signals equal to corresponding fluorescence lifetimes. However, it is impossible to visually distinguish the 3 ns time-delay with the span of 5 μs in Figure 3. The different intensity profiles for different lifetimes are more likely to overlap in time axis.

### 2.3. Time-Delay Estimation

In the process of cytometric measurement, the light pulse signals *L_fs_*(*t*) and *L_fl_*(*t*) are converted to electrical signals by photovoltaic detectors and then processed by electronic systems. The final output signals corresponding to *L_fs_*(*t*) and *L_fl_*(*t*), defined as *fs* and *fl*, are the eventually handled using time-delay estimation. Therefore, the time-delay acquired includes fluorescence lifetime and the time-delay introduced by hardware. In order to distinguish the lifetime, calibration process should be applied to eliminate the time-delay introduced by photovoltaic conversion and electric systems. The calibration process could be found in Section 3.2. With the purpose of distinguish the lifetime (nanosecond) from the acquired time-delay (microsecond), there are two key points should be confirmed: (1) The time-domain resolution of the method described in manuscript (nanosecond could be recognized); (2) The calibration process is effective and accurate (The time-delay introduced by photovoltaic conversion and electric systems could be eliminated accurately).

**Figure 4:**
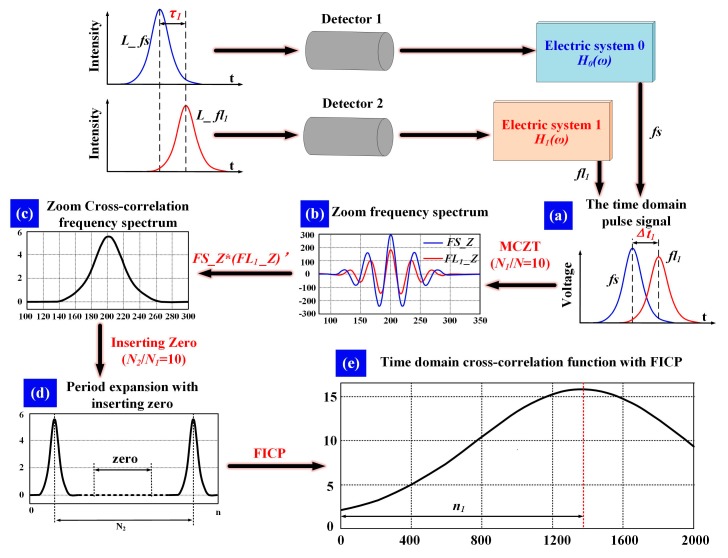
Steps of time-delay estimation between *fs* and *fl*_1_. (**a**) Pulse signals of *fs* and *fl*_1_ after photovoltaic conversion and electronic systems; (**b**) Zoom frequency spectrum of *fs*(*n*) and *fl*_1_(*n*) calculated with MCZT, *N*_1_*/N* = 10; (**c**) Cross-correlation frequency spectrum calculated with *FS_Z* and *FL*_1_*_Z*; (**d**) Cross-correlation frequency spectrum period expansion by inserting zero, *N*_2_*/N*_1_ = 10; (**e**) Time domain cross-correlation function calculated with FICP. (Δ*t*: time-delay between *fs* and *fl*_1_; *n*_1_: the time-delay estimation result).

To estimate the time-delay between *fs* and *fl*, the cross-correlation function is applied to restrain the influence of noise. The MCZT [25] method is executed to acquire the spectral information with high resolution. The FICP [26] algorithm is then used to improve the resolution of the time-domain cross-correlative function. The time-delay estimation processing using the comprehensive method presented in this work is illustrated in Figure 4.

Assuming that the forward-scattered pulse signal *fs*(*n*) and the fluorescence pulse signal *fl_m_*(*n*) are data sequences containing *N* points, the corresponding zoomed frequency spectrum is described by the following transform:

(4) $$\{\begin{array}{c} FS(k)=MCZT(fs(n))=\sum_{n=0}^{N-1}fs(n)\exp(-\frac{j2\pi }{N_{1}}kn)\\ FL_{m}(k)=MCZT(fl_{m}(n))=\sum_{n=0}^{N-1}fl_{m}(n)\exp(-\frac{j2\pi }{N_{1}}kn) \end{array}$$

where *m* = 1, 2, 3 and *k* = 0, 1 … *N* − 1.

The sampling frequency is raised to *N*_2_/*N*_1_ times that of the initial sampling frequency of analog-to-digital converter (ADC), when the cycle of frequency spectrum is extended from *N*_1_ to *N*_2_ by inserting zero. The resolution of the time-domain cross-correlation function is increased to *N*_2_/*N*_1_ times with the same number of calculations. The complete cross-correlation frequency spectrum is constructed as follows:

(5) $$R_{m}(k)=\{\begin{array}{c} R_{1}(k)=FS(k)FL_{m}^{*}(k),\\ 0\\ R_{1}^{*}(N_{2}-k) \end{array}\begin{array}{c} k=0,1,\cdots ,N-1\\ k=N,\cdots ,N_{2}-N\\ k=N_{2}-N+1,\cdots ,N_{2}-1 \end{array}$$

where FL_m_^*^ (*k*) is the conjugation of *FL_m_*(*k*)*, R*_1_^*^ (*N*_2_* − k*) is the conjugation of *R*_1_ (*N*_2_* − k*)*.*

The time-delay between *fs* and *fl* falls into a certain limited range. The first part *r_m_*_1_(*n*) and the last part *r_m_*_2_(*n*) of the cross-correlation function in the time domain are calculated by Equations (6) and (7) respectively, as follows:

(6) $$r_{m1}(n)=[\sum_{k=0}^{N-1}R_{1}(k)\exp(j\frac{2\pi }{N_{2}}kn)+\exp(-j\frac{2\pi }{N_{2}}Nn)\sum_{k=0}^{N-1}R_{1}^{*}(N-k)\exp(j\frac{2\pi }{N_{2}}kn)]/N_{2}$$

(7) $$\begin{array}{ll} r_{m2}(n) & =\exp(j\frac{2\pi }{N_{2}}Nn)\sum_{k=0}^{N-1}\left[R_{1}(k)\exp(-j\frac{2\pi }{N_{2}}kn\right)]/N_{2}\\ & +\exp[j\frac{2\pi }{N_{2}}(N^{2}-nN)]\sum_{k=0}^{N-1}\left[R_{1}^{*}(N-k)\exp(-j\frac{2\pi }{N_{2}}kn\right)]\exp(j\frac{2\pi }{N_{2}}kn)/N_{2} \end{array}$$

Eventually, the integrated cross-correlation function in the time domain *r_m_*(*n*) is constructed with *r_m_*_1_(*n*) and *r_m_*_2_(*n*) as:

(8) $$r_{m}(n)=\{\begin{array}{cc} r_{m1}(n), & n=0,1,\cdots ,N-1\\ r_{m2}(2N+n) & n=-N,\cdots ,-1 \end{array}$$

## 3. Materials and Method

### 3.1. Time-Delay Estimation with Microspheres

The sample used in this study included Cyto-Trol Control Cells Kit (Beckman Coulter Inc. Ref. 6604248) and fluorochrome-conjugated antibodies CD3-FITC (Beckman Coulter Inc. Ref. A07746), CD8-PE (Beckman Coulter Inc. Ref. A07757) and CD4-PC5 (Beckman Coulter Inc. Ref. A07752). They are lyophilized preparation of human lymphocytes that exhibit surface antigens detectable with monoclonal antibodies. These cells are directly conjugated with monoclonal antibodies. All fluorochrome-conjugated antibodies are excited at 488 nm. The PMTs used for the detection of fluorescence light are H11903-20mod (Hamamatsu) with a frequency bandwidth from DC to 1 MHz and a luminous sensitivity of 500 μA/lm. The photodiode used for the detection of forward-scattered light is Si Pin photodiode S9195 (Hamamatsu) with a terminal capacitance of 80 pF and a photo sensitivity of 0.28 A/W (*λ* = 405 nm). The ADCs used are AD9690 (500 MSPS: Mega samples per second, 14 bit, ANALOG DEVICE) and AD9446-100 (100 MSPS, 14 bit, ANALOG DEVICE). The function generator used for time-delay calibration is a dual channel arbitrary/function generator AFG3022C (Tektronix) with a sample rate of 250 MSPS and a bandwidth of 25 MHz.

The lymphocytes were stained with three-color reagents simultaneously and used to acquire the cytometric pulses (*fs*, *fl*_1_, *fl*_2_ and *fl*_3_). The flow cytometry measurements were executed using a Gallios Flow Cytometer (Beckman Coulter Corp., Brea, CA, USA).

### 3.2. Time-Delay Calibration of Photovoltaic Conversion and Electric Systems

**Figure 5:**
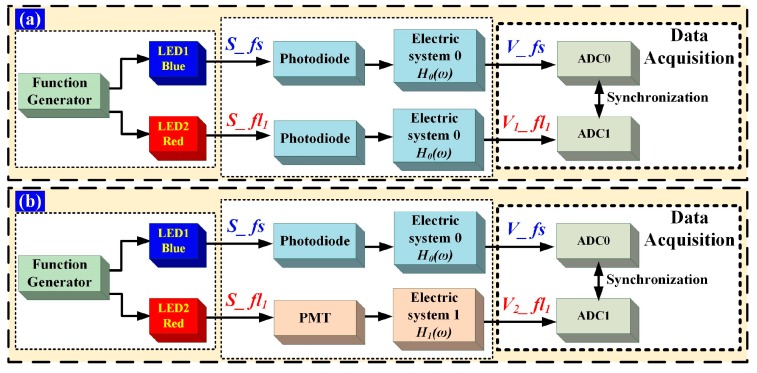
Time-delay calibration. The LED1 emission light is blue and the LED2 emission light is red; *S_fs* is forward-scattered light pulse, *S_fl*_1_ is the fluorescence light pulse. (**a**) Light-pulse time-delay calibration. *H*_0_(*ω*) is the frequency-domain transfer function of electric system 0; (**b**) Hardware time-delay calibration. *H*_1_(*ω*) is the frequency-domain transfer function of electric system *1*.

As mentioned above, the time-delay acquired includes fluorescence lifetime and the time-delay introduced by hardware (includes photovoltaic conversion and electric system). It is necessary to account for the inherent time-delay introduced by the hardware variables, such as the differences in the photoelectric detector, circuitry and cable, via calibration. Generally, the electrical hardware system used in flow cytometer is time invariant. Namely, the transfer function of electrical hardware system is time-independent, which means that the time-delay introduced by hardware could be acquired and eliminated accurately. The calibration process is performed as shown in Figure 5. 

Two light-emitting diodes (LEDs) are pulsed by AFG3022C with Gaussian waveforms as light sources. *S_fs* and *S_fl*_1_ are Gaussian-shaped light pulses. *S_fs* is used to represent the forward-scattered light pulse and *S_fl*_1_ is used to represent the fluorescent-light pulse of a flow cytometer. *S_fs* and *S_fl*_1_ are the emissions of LED1 and LED2, which are separately pulsed by the two channels of AFG3022C. The corresponding electrical pulse signal is presented in terms of *V_fs* and *V*_1_*_fl*_1_ (*V*_2_*_fl*_1_) and then converted to digital signals using ADCs labeled ADC0 and ADC1, respectively. The ADC0 and ADC1 clock signals are provided by one crystal oscillator, to ensure that *V_fs* and *V*_1_*_fl*_1_ (*V*_2_*_fl*_1_) are converted simultaneously. 

Two steps are taken for the calibration of light-pulse and hardware time-delay, respectively. Figure 5a shows the calibration of light-pulse time-delay, to ensure that *S_fs* and *S_fl*_1_ are synchronous. *S_fs* and *S_fl*_1_ are detected by the same photodiodes, processed with the same electric system 0 and then conserved by ADC0 and ADC1, respectively. The time-delay between *V_fs* and *V*_1_*_fl*_1_ is evaluated and the phase shift between the two channels of AFG3022C adjusted until the time-delay between *V_fs* and *V*_1_*_fl*_1_ is zero. So far, the time-delay error between the two channels of function generator and the response time differences of the two LEDs has been calibrated. *S_fs* and *S_fl*_1_ are synchronous. As illustrated in Figure 5b, *S_fl*_1_ is detected by a PMT and processed with electric system 1. The PMT and electric system 1 are the same as those used in actual cytometric measurement. The time-delay between *V_fs* and *V*_2_*_fl*_1_, which is the time-delay introduced by the photovoltaic conversion and electric systems can be acquired.

## 4. Results and Discussion

### 4.1. Time-Delay Estimation

**Figure 6:**
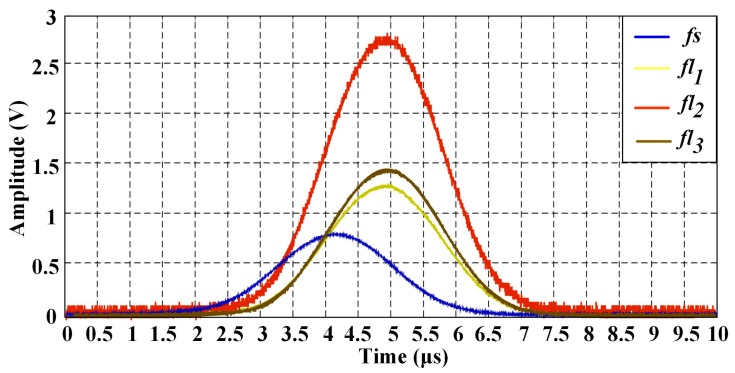
Cytometric pulse signals from single cell stained with three-color reagents simultaneously. Four AD9446-100 (100 MSPS) are used for the analogy-to-digital conversation.

The cells used for cytometric measurement are labeled with three-color reagents simultaneously; therefore, a series of pulse signals (*fs*, *fl*_1_, *fl*_2_ and *fl*_3_) from one single cell could be acquired. One series of pulse signals is taken as an example in Figure 6. The ADC used in measurement is AD9446-100, with a conversion rate of 100 MSPS. The time-delay between the *fl*_1_, *fl*_2_, *fl*_3_ and *fs* was estimated using the FICP algorithm. The series of signals were converted from analog to digital by ADCs with a higher conversion rate (AD9690) and the peak locations of these pulse signals were acquired using a Gauss fitting algorithm, for comparison with the results of the proposed method. 

**Figure 7:**
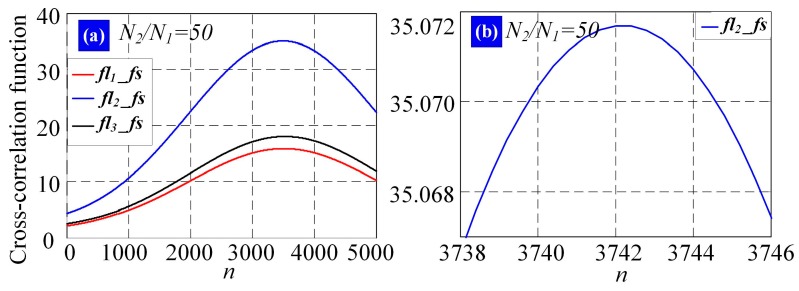
(**a**) Cross-correlation function of *fs*(*n*) and *fl_m_*(*n*) in time-domain calculated with FICP (*N*_2_/*N*_1_ = 50). The red curves are the cross-correlation functions of *fs*(*n*) and *fl*_1_(*n*); the blue curves are the cross-correlation functions of *fs*(*n*) and *fl*_2_(*n*); the black curves are the cross-correlation functions of *fs*(*n*) and *fl*_3_(*n*); (**b**) Peak detail of cross-correlation function of *fs*(*n*) and *fl*_2_(*n*) in (**a**).

The sampling frequency of *fs* and *fl_m_* in this work is 100 MHz, meaning that the time interval between adjacent points in the pulse signal is 10 ns. The time-domain cross-correlation function *r*(*n*) is calculated with FICP (*N*_2_*/N*_1_ = 50), as shown in Figure 7. Only the points around the peak are acquired and the time resolution is 0.2 ns (10 *ns*/(*N*_2_/*N*_1_)); the corresponding time-delay is Δ*t*_1_ = 765.6 ns (3828 × 0.2 ns), Δ*t*_2_ = 748.4 ns (3742 × 0.2 ns) and Δ*t*_3_ = 772.6 ns (3863 × 0.2 ns). The resolution of the cross-correlation peak is improved by increasing the interpolation multiple of the frequency spectrum, namely, the computational accuracy of the time-delay estimation is higher with FICP, particularly for the pulse signals of low sampling frequency.

### 4.2. Curve Fitting

**Figure 8:**
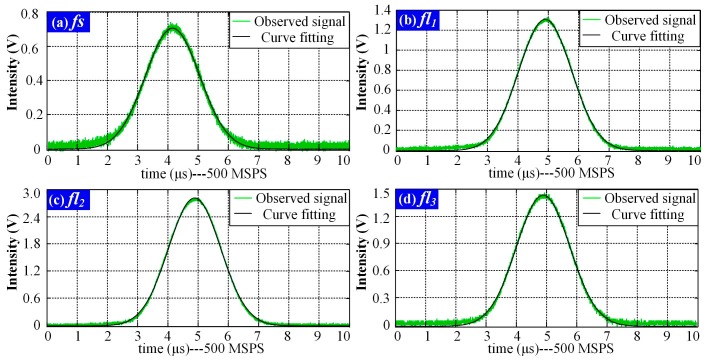
Gauss fitting results of pulse signals (*fs*, *fl*_1_, *fl*_2_ and *fl*_3_). (**a**) Results of pulse signal *fs*; (**b**) Results of pulse signal *fl*_1_; (**c**) Results of pulse signal *fl*_2_; (**d**) Results of pulse signal *fl*_3_.

One series of pulse signals (*fs*, *fl*_1_, *fl*_2_ and *fl*_3_) was taken as an example for the FICP feasibility analysis. The curves obtained with Gauss fitting are shown in Figure 8, where the green curves are the observed signals and the black curves are the Gauss fitting results.

The Gauss fitting was modeled by $K_{1}e^{-({\left(t-K_{2}\right)/K_{3})}^{2}}$ and the results were evaluated with the root mean square error (*RMSE*) and the *R-squared* values. *RMSE* is an estimate of the standard deviation of the random component in a data set and was used to evaluate the deviation between the curve fitting result and the observed signal in this study. The *RMSE* is defined as

(9) $$RMSE=\sqrt{\frac{1}{n}\sum_{i=1}^{n}{\left(X_{obs,i}-X_{model,i}\right)}^{2}}$$

where *X_obs_* is the observed value, *X_fit_* is the curve fitting result and *n* is the length of the observed signal, which is equal to that of the fitting result.

*R-squared* is the square of the correlation between the observed and predicted values, defined as the ratio of the sum of the squares of the regression (*SSR*) and the total sum of squares (*SST*). 

(10) $$R-squared=\frac{SSR}{SST}$$

*SSR* and *SST* are defined as $SSR=\frac{1}{n}\sum_{i=1}^{n}{\left(X_{fit,i}-{\bar{X}}_{obs}\right)}^{2}$ and $SST=\sum_{i=1}^{n}{\left(X_{obs,i}-{\bar{X}}_{obs}\right)}^{2}$, where ${\bar{X}}_{obs}$ is the mean value of the signal. The Gauss fitting results at conversion rates of 500 MSPS and 100 MSPS, together with the evaluation indexes are listed in Table 1. As indicated, the *RMSE* is less than 0.03 and the *R-squared* values are larger than 0.99. Therefore, the pulse signals were represented nearly perfectly by the Gauss fitting results. 

Table 2 lists the peak locations and the time -delays Δ*t*_1_, Δ*t*_2_ and Δ*t*_3_ of cytometric pulse signals calculated using Gauss fitting and the time-delays Δ*t*_1_, Δ*t*_2_ and Δ*t*_3_, acquired with FICP. As illustrated, the time-delays of each fluorescence pulse signals obtained via Gauss fitting and FICP are very close. The FICP algorithm is feasible for time-delay estimation.

Then, 5000 series pulse signals of fluorescently labeled cells were detected and the time delays between *fl*_1_, *fl*_2_, *fl*_3_ and *fs* were calculated using Gauss fitting and the FICP method. Table 3 lists the statistical results of the time-delay estimation after calibration. The mean values, standard deviations (SDs,$\sigma =\sqrt{\frac{1}{n-1}\sum_{i=1}^{n}{\left(x_{i}-\bar{x}\right)}^{2}}$) and relative standard deviations (RSDs, $\frac{\sigma }{\bar{x}}\times 100\text{\%}$) were taken as indexes to evaluate the statistical properties of the fluorescence pulse signals obtained from the same type of fluorochrome.

As can be seen, the mean values of each lifetime obtained via Gauss fitting and FICP are very close and the SDs and RSDs determined for the FICP method values are significantly smaller than those for the Gauss fitting results. The calculated lifetimes *τ*_1_, *τ*_2_ and *τ*_3_, are close to the reference values 7.5 ns, 5.0 ns and 10.2 ns, which are provided by the vendor. Therefore, the lifetime can be detected correctly using the proposed method.

### 4.3. Verification with Pulse Width Variation

As mentioned in Section 2.2, the width of fluorescence pulse signal will be influenced by fluorescence lifetime and the threshold in the detection. The width variation could be used for the verification of lifetime measurement accuracy. 

Considering the process of detection, the observed fluorescence pulse signals could be expressed as

(11) $$\{\begin{array}{c} fs(t)*(N_{1}P_{1}e^{-\frac{t}{\tau_{1}}})h\frac{c}{\lambda_{1}}G_{1}a_{1}=fl_{1}(t)\\ fs(t)*(N_{2}P_{2}e^{-\frac{t}{\tau_{2}}})h\frac{c}{\lambda_{2}}G_{2}a_{2}=fl_{2}(t)\\ fs(t)*(N_{3}P_{3}e^{-\frac{t}{\tau_{3}}})h\frac{c}{\lambda_{3}}G_{3}a_{3}=fl_{3}(t) \end{array}$$

where *N*_1_, *N*_2_ and *N*_3_ are the number of fluorochromes labeled on a single cell; *P*_1_, *P*_2_ and *P*_3_ are the corresponding quantum efficiencies; *λ*_1_, *λ*_2_ and *λ*_3_ are the average wavelength of each fluorescence emission spectrum; *c* is the velocity of light; *h* is the Planck’s constant; *G*_1_, *G*_2_ and *G*_3_ are the gains of each circuit system (include the gains of photovoltaic conversion and electric system); *a*_1_, *a*_2_ and *a*_3_ are the luminous sensitivity of each detectors at *λ*_1_, *λ*_2_ and *λ*_3_. In the measurement, *fl*_1_(*t*), *fl*_2_(*t*) and *fl*_3_(*t*) are the observed results, *λ*_1_, *λ*_2_, *λ*_3_, *a*_1_, *a*_2_, *a*_3_, *G*_1_, *G*_2_ and *G*_3_ are known parameters. Equation (11) could be manipulated as

(12) $$\{\begin{array}{c} N_{1}P_{1}fs(t)*(e^{-\frac{t}{\tau_{1}}})=fl_{1}(t)/（h\frac{c}{\lambda_{1}}G_{1}a_{1}）\\ N_{2}P_{2}fs(t)*(e^{-\frac{t}{\tau 2}})=fl_{2}(t)/（h\frac{c}{\lambda_{2}}G_{2}a_{2}）\\ N_{3}P_{3}fs(t)*(e^{-\frac{t}{\tau_{3}}})=fl_{3}(t)/（h\frac{c}{\lambda_{3}}G_{3}a_{3}） \end{array}$$

In order to analyze the individual influence of lifetimes (fs(t)∗(e−tτ)), *N*_1_*P*_1_, *N*_2_*P*_2_ and *N*_3_*P*_3_ are normalized to acquire the relative relationship between width variations of different lifetimes, as follows:

(13) $$\{\begin{array}{c} \frac{N_{1}P_{1}}{N_{2}P_{2}}=\frac{fl_{1}(0)G_{2}a_{2}\lambda_{1}}{fl_{2}(0)G_{1}a_{1}\lambda_{2}}\\ \frac{N_{1}P_{1}}{N_{3}P_{3}}=\frac{fl_{1}(0)G_{3}a_{3}\lambda_{1}}{fl_{3}(0)G_{1}a_{1}\lambda_{3}} \end{array}$$

where *fl*_1_(0), *fl*_2_(0) and *fl*_3_(0) are the baseline values of *fl*_1_(*t*), *fl*_2_(*t*) and *fl*_3_(*t*), namely, the initial intensities at time 0.

**Figure 9:**
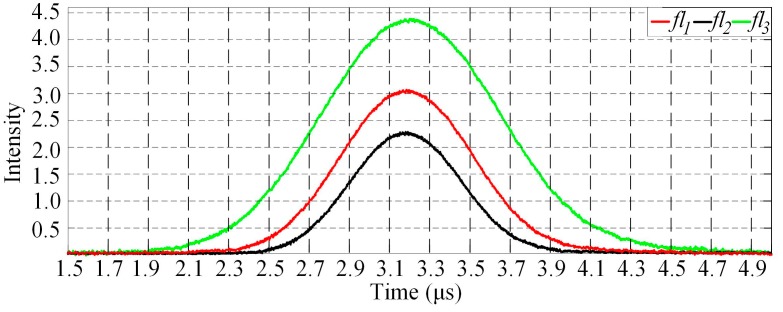
Fluorescence pulse signals after normalization. The intensity is used to indicate the relative relationship with no units.

The curves of Figure 6 are normalized and the results are shown in Figure 9. As illustrated, the baselines of normalized pulse signals are almost equal. The peak value of pulse signal is lower with shorter lifetime. Considering the influence of threshold, the pulse width of the signal with smaller lifetime is shorter and the pulse width variation compared to the width of fs is larger. In order to illustrate the width variation, the parameter *K*_3_ of Gauss fitting ($K_{1}e^{-({\left(t-K_{2}\right)/K_{3})}^{2}}$) is used in this work. Analyzing the normalized pulse signals with Gauss fitting, the width variations of *fl*_1_, *fl*_2_ and *fl*_3_ represented by Δ*K*_3_ are 16.62, 19.60 and 9.47, respectively.

**Figure 10:**
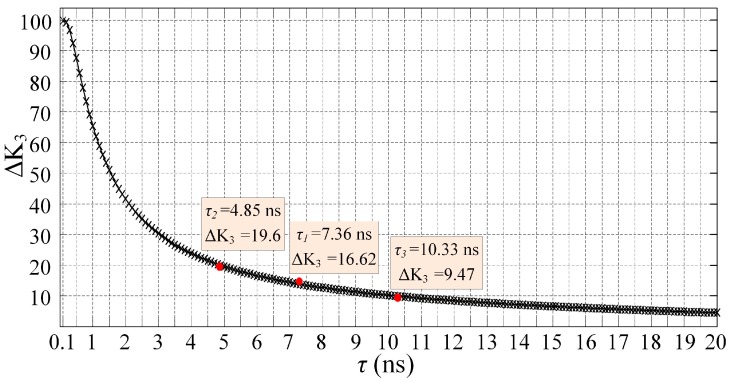
Calculated pulse width variation with different lifetimes. The blank curve marked with “x” represents the results acquired using Equation (2). The red points are the results of proposed method in this work.

On other hand, the width variation of different lifetimes could be acquired using Equation (2). The pulse signal fs in Figure 6 is used as *L_fs_*(*t*), the lifetime *τ* ranges from 0.1 ns to 20 ns, stepped by 0.1 ns. The relationship between lifetime and width variation could be acquired and the results of Δ*K*_3_ are shown in Figure 10 for the verification of lifetime measurement accuracy using the proposed method. As shown, the width variations acquired with the proposed method in this work are consistent with those from the theoretical calculations.

## 5. Conclusions

To summarize, this work reports a simple method of measurement of fluorescence lifetime based on cytometric pulse signals’ time-delay estimation. The lifetime is acquired by estimating the pulse signals’ time-delay and then calibrating the time-delay of hardware. A high-resolution time-delay estimation method combining MCZT with FICP, in order to characterize the fluorescence pulse signal of a standard flow cytometer without changing the hardware, is applied. The feasibility of the time-delay estimation is verified by Gauss fitting. Finally, the statistical results of the proposed method are compared with reference values provided by the vendor. Meanwhile, the measured lifetimes are verified with the calculated pulse width variation. The proposed method could be applied to any past or presently marketed instrument and it could involve measuring the fluorescence dynamics with multiple fluorophores. The fluorescence lifetime contains and can reveal, more information than the traditional data obtained by peak-area-width list mode. Future work will involve expanding to cell sorting systems and using this approach to alleviate intensity-related problems such as spectral overlap and auto-fluorescence noise. 

## Figures and Tables

**Table 1:** Results of Gauss fitting and evaluation indexes.

Signals	Conversion Rate (MSPS)	Curves Fitting Results			Evaluation Indexes	
		*K*_1_	*K*_2_	*K*_3_	*RMSE*	*R-Squared*
*fs*	500	0.7357	2065	639.3	0.0201	0.9928
	100	0.7386	412.3	128.9	0.0201	0.9928
*fl_1_*	500	1.309	2446	615.8	0.0182	0.9981
	100	1.308	488.3	123	0.0181	0.9982
*fl_2_*	500	2.901	2440	611.6	0.0290	0.9990
	100	2.898	486.9	122	0.0276	0.9991
*fl_3_*	500	1.475	2453	625.9	0.0288	0.9963
	100	1.475	489.5	125.2	0.0286	0.9964

**Table 2:** Results of time-delay estimation.

		Peak Location (ns)				Time-Delay (ns)		
		*fs*	*fl*_1_	*fl*_2_	*fl*_3_	Δ*t*_1_	Δ*t*_2_	Δ*t*_3_
FICP		----	----	----	----	765.6	748.4	772.6
Curve fitting	500 MSPS	4130	4892	4880	4906	762	750	776
	100 MSPS	4123	4883	4869	4895	760	746	772

**Table 3:** Statistical results of fluorescence lifetime.

		τ_1_			τ_2_			τ_3_		
		Mean (ns)	SD (ns)	RSD (%)	Mean (ns)	SD (ns)	RSD (%)	Mean (ns)	SD (ns)	RSD(%)
FICP		7.36	1.58	18.90	4.85	1.16	23.92	10.33	1.65	15.97
Curve fitting	500 MSPS	7.24	1.76	21.36	4.92	1.24	25.20	10.41	1.88	18.06
	100 MSPS	7.17	1.93	23.62	5.11	1.59	31.12	11.54	2.13	18.46

## Abbreviations

FCM	Flow cytometry
ADC	Analog-to-Digital Converter
MCZT	Modified Chirp Z-Transform
FICP	Fine Interpolation of Correlation Peak
PMT	Photomultiplier tube
LED	Light-emitting diodes
MSPS	Mega samples per second
RMSE	Root Mean Square Error
SSR	Sum of Squares of the Regression
SST	Total Sum of Squares
SD	Standard Deviation
RSD	Relative standard deviation
CV	Coefficients of Variation
